# Improving the efficiency of feed utilization in poultry by selection. 2. Genetic parameters of excretion traits and correlations with anatomy of the gastro-intestinal tract and digestive efficiency

**DOI:** 10.1186/1471-2156-12-71

**Published:** 2011-08-17

**Authors:** Hugues de Verdal, Agnès Narcy, Denis Bastianelli, Hervé Chapuis, Nathalie Même, Séverine Urvoix, Elisabeth Le Bihan-Duval, Sandrine Mignon-Grasteau

**Affiliations:** 1 INRA, UR83 Recherches Avicoles, F-37380, Nouzilly, France; 2 CIRAD, UMR SELMET, 34398 Montpellier cedex 5, France; 3 SYSAAF, UR83 Recherches Avicoles, F-37380, Nouzilly, France

## Abstract

**Background:**

Poultry production has been widely criticized for its negative environmental impact related to the quantity of manure produced and to its nitrogen and phosphorus content. In this study, we investigated which traits related to excretion could be used to select chickens for lower environmental pollution.

The genetic parameters of several excretion traits were estimated on 630 chickens originating from 2 chicken lines divergently selected on apparent metabolisable energy corrected for zero nitrogen (AMEn) at constant body weight. The quantity of excreta relative to feed consumption (CDUDM), the nitrogen and phosphorus excreted, the nitrogen to phosphorus ratio and the water content of excreta were measured, and the consequences of such selection on performance and gastro-intestinal tract (GIT) characteristics estimated. The genetic correlations between excretion, GIT and performance traits were established.

**Results:**

Heritability estimates were high for CDUDM and the nitrogen excretion rate (0.30 and 0.29, respectively). The other excretion measurements showed low to moderate heritability estimates, ranging from 0.10 for excreta water content to 0.22 for the phosphorus excretion rate. Except for the excreta water content, the CDUDM was highly correlated with the excretion traits, ranging from -0.64 to -1.00. The genetic correlations between AMEn or CDUDM and the GIT characteristics were very similar and showed that a decrease in chicken excretion involves an increase in weight of the upper part of the GIT, and a decrease in the weight of the small intestine.

**Conclusion:**

In order to limit the environmental impact of chicken production, AMEn and CDUDM seem to be more suitable criteria to include in selection schemes than feed efficiency traits.

## Background

Animal excreta provide valuable organic fertilizers. However, in regions where they are used in excess, they can be associated with environmental pollution [1], such as nitrate contamination, soil acidification and water eutrophication. This is often the case for poultry production in Europe, due to the high concentration of poultry farms in several regions such as Brittany in France. For example, French poultry meat production was estimated to be 2.0 10^6^t in 2005 and the quantity of faeces generated has been estimated at 3.0 10^6^t for manure and 6.0 10^6^t for excreta and liquid manure [2].

Nitrogen (N) and phosphorus (P) concentrations in poultry manure are two major issues [3,4]. P is partly present in poultry diets as phytic phosphorus, i.e. a form that is poorly digestible for birds due to a lack of the adequate endogenous phytase activity [3]. As a consequence, the amount of P excreted represents up to 60 or even 80% of P intake [5]. The problem of N mainly originates from the difference between the amino-acid (AA) composition of the diet and the ideal AA profile for broilers. Meeting animal requirements therefore involves increasing the protein content of the diet, and thus N excretion [6]. The common approach to solve these problems is either using synthetic amino acids to limit the protein content of a diet or supplementing the feed with phytase to improve P absorption, which could limit N and P excretion by birds [3,7].

In addition to nutritional methods to reduce poultry excretion, genetic solutions can also be sought. Indeed, several experiments have shown that selection could make a significant contribution to reduction in poultry excretion. For example, using divergent selection on phytate phosphorus bioavailability over 3 generations, Zhang et al. [8] obtained a difference of 9.7% between the high and low lines selected on their capacity to retain P and chickens of the low line showed an improvement of BW and FCR compared to the high line [9].

Similarly, Mignon-Grasteau et al. [10] created the D+ and D- chicken lines by a divergent selection experiment based on high or low ability to digest a poor variety of wheat, respectively, at constant BW. Digestive efficiency was assessed by apparent metabolisable energy corrected for zero nitrogen retention (AMEn). Selection was made at 3 weeks of age. At the 7^th ^generation of selection, D+ birds showed more favourable values than D- birds for AMEn (3258 vs 1916 kcal.kgMS^-1^, respectively) or FCR (1.70 vs 3.13, respectively). Both lines had similar BW at 21 d (399 vs 394 g for D+ and D-, respectively) and at 53 d (1943 vs 1903 g for D+ and D-, respectively, [11]. In addition to the wide differences in digestive capacity obtained between these lines, de Verdal et al. [12] showed that the gastro-intestinal tract had been extensively modified by the selection process. More recently, Mignon-Grasteau et al. [13] showed that D- birds excreted 36.6% more than the D+ birds, and that the difference was even greater for P (+52.5% for D- birds). However, the genetic relationships between the selection criterion (AMEn) and the traits modified by selection (morphology and excretion) remain to be established. Furthermore, it would be interesting to compare the impact on the excretion characteristics of selection on the usual selection criterion of feed efficiency (FCR) or on digestive efficiency (AMEn). It can be expected that responses will not be the same since FCR is related to a broad range of traits including feed consumption, tissue deposition, heat production due to basal metabolic intensity, digestion or to physical activity and efficiency in converting of feed [14] whereas AMEn is more closely linked to digestive efficiency.

The aim of the present study was first to estimate the genetic parameters of the excretion traits in these two divergent lines, second to estimate the genetic correlations between excretion traits, growth performance and gastro-intestinal tract (GIT) morphology and, finally, to evaluate which criteria could be used to select against chicken excretion, including excretion of N and P, without any significant impact on growth performance.

## Methods

### Birds and housing

The experiment was conducted according to the guidelines of the French Ministry of Agriculture for Animal Research, and included 630 birds (307 males and 323 females) of the 8^th ^generation of selection of D+ and D- lines, reared in 3 hatches, each separated by 4 weeks. The pedigree file included animals from all the generations of selection (i.e., 4495 birds). They were individually weighed at hatching and placed in groups of 4 or 5 chicks in metal cages (36 cm long × 22 cm wide × 40 cm high) for 3 d. After 3 d, chicks were randomly allocated to individual cages, in 3 different rearing rooms. The environmental conditions were controlled for ventilation, lighting program (24 L: 0D from 1 d to 7 d and 23 L: 1D from 8 d to 23 d, dark periods beginning at midnight) and temperature (from 33°C at 1 d to 22°C at 23 d). Mortality was recorded daily. The birds had free access to water and food. They were fed a wheat-based diet similar to that used by de Verdal et al. [15].

### Growth and excretion traits

All birds were individually weighed at 17 (BW17) and 23 d (BW23) of age. The weight gain between 17 and 23 d was calculated (WG). Individual total feed intake (FI) was recorded from 17 to 23 d and feed conversion ratio (FCR) was calculated. Excreta were collected individually between 17 and 23 d, using the method of individual total collection of excreta [16]. Total excreta were weighed and dried to obtain both fresh excreta weight (FEW) and dry excreta weight (DEW). The water content of excreta (WE) was calculated as (FEW-DEW)/FEW. The fresh and dry excreta weights relative to body weight (FEW/BW and DEW/BW, respectively) were calculated. AMEn, nitrogen excretion (NE) and nitrogen consumption (NI) were measured for all birds using Near Infrared spectrophotometry (NIRS, Foss spectrometer NIRSystems 6500, Inc., Silver Spring, MD), according to the method of Bastianelli et al. [17] after validating and updating calibration equations with 20 reference measurements. AMEn was calculated according to the equation described by Lessire [18]:

(1) EMAn=1∕C.[C.Ba-E.Be-(C.Na∕100-E.S∕100.NT∕100).34406]

where C is the feed intake in dry mass (MS) (g), Ba the gross energy of the diet (J.g^-1^), Na the total nitrogen concentration of the diet (%), E the lyophilized excreta weight (g), S the MS proportion in the lyophilized excreta (%), Be the gross energy of the lyophilized excreta (J.g^-1^), and NT the total nitrogen concentration in the excreta (%).

Phosphorus excretion (PE) and consumption (PI) were determined according to the Vanadate colorimetric method using a Phosphorus UV-kit (BioMérieux SA, Lyon, France). The NE/PE and FEW/FI ratios, the coefficient of digestive use of dry matter (CDUDM = 100 - (DEW/FI.100), NE/NI and PE/PI ratios were calculated. NE/PE can be viewed as an indicator of individual environmental performances whereas NE/NI and PE/PI are rather biological indicators of individual capacities to retain N and P. The residual feed intake (RFI) was calculated as the difference between the observed feed consumption and its estimate obtained by linear regression on metabolic BW (BW^0.75^) and weight gain (BWG) between 17 and 23 d [19].

### Morphology of digestive tract

At 23 d of age, after overnight fasting (8 h), all chicks were sacrificed by CO_2 _inhalation. The crop, proventriculus and gizzard were excised and weighed (CRW, PRW, and GZW, respectively). The duodenum (from pylorus to pancreatic loop), jejunum (from the pancreatic loop to Meckel's diverticulum), and ileum (from Meckel's diverticulum to the ileo-caecal junction) were sampled and their lengths measured (DL, JL, and IL, respectively). Segments were then cleaned and weighed (DW, JW, and IW, respectively). The weight to length ratio of each segment (DD, JD, and ID, respectively) was also calculated as an indicator of intestine density [20]. All the data regarding organ weight and length were expressed per kg of BW.

### Statistical analyses

All data were analyzed according to the General Linear Models (GLM) procedure of SAS [21]. For all traits, the following model was used:

(2) $${\text{y}}_{\text{ijkl}}=\mu +{\text{L}}_{\text{i}}+{\text{C}}_{\text{j}}+{\text{H}}_{\text{k}}+{\text{S}}_{\text{l}}+{\text{e}}_{\text{ijkl}}$$

where y_ijkl _is the performance of animal m, µ the general mean, L_i _the fixed effect of line i (i = D+ or D-), C_j _the effect of rearing room j (j = 1 to 3), H_k _the fixed effect of hatch k (k = 1 to 3), S_l _the fixed effect of sex l, and e_ijkl _the residual term for animal l. Least square means and standard deviations were estimated for D+ and D- lines for each trait. Differences were considered significant when the *P*-value was lower than 0.05.

### Estimation of genetic parameters

Genetic parameters were estimated by the REML (REstricted Maximum Likelihood) method with the VCE4 software [22]. For all traits except BW23, FCR, GZW PRW and CDUDM, the model [3] was used. As preliminary analyses indicated the presence of a significant maternal effect for BW23, FCR, GZW, PRW and CDUDM, these traits were analyzed with model [4].

(3) $${\text{y}}_{\text{ijklm}}=\mu +{\text{L}}_{\text{i}}+{\text{C}}_{\text{j}}+{\text{H}}_{\text{k}}+{\text{S}}_{\text{l}}+{\text{a}}_{\text{m}}+{\text{e}}_{\text{ijklm}}$$

(4) $${\text{y}}_{\text{ijklm}}=\mu +{\text{L}}_{\text{i}}+{\text{C}}_{\text{j}}+{\text{H}}_{\text{k}}+{\text{S}}_{\text{l}}+{\text{a}}_{\text{m}}+{\text{d}}_{\text{n}}+{\text{e}}_{\text{ijklmn}}$$

with a_m _the random additive genetic effect of the animal m (N = 4495) and d_n _the maternal permanent environmental effect. The pedigree file included animals from the 8 generations of the selection experiment which have been all recorded for BW23, FCR, AMEn and CDUDM. All these data have been included in the analyses. Anatomy and excretion traits were recorded only for the last generation (N = 630). As several traits presented very strong genetic correlations, it was not possible to run a single analysis including all traits, meaning that distinct multi-trait analyses were performed. In order to avoid bias in estimates due to the effect of selection in our lines, all analyses included selection criteria, i.e. AMEn and BW23. Each analysis also included two other traits, to be able to estimate genetic correlations between all traits. A total of 169 analyses were thus performed with 4 traits each time: BW23, AMEn and two others traits. The parameter estimates and the standard errors presented were the average of the estimates obtained in the various analyses. Standard errors were not available for several analyses, as several traits presented very high correlations and/or low heritability estimates, preventing the maximum likelihood algorithm from reaching a single optimum.

The following equations were used to compare the expected direct (CR_(Y.Y)_, equation [5]) and indirect (CR_(Y.X)_, equation [6]) correlated response to selection on the different criteria:

(5) $$\text{C}{\text{R}}_{\left(\text{Y}\text{.Y}\right)}={\text{i}}_{\text{Y}}\times {{\text{h}}^{2}}_{\text{Y}}\times \sigma {\text{p}}_{\text{Y}}$$

(6) $$\text{C}{\text{R}}_{\left(\text{Y}\text{.X}\right)}={\text{i}}_{\text{x}}\times \surd \left({{\text{h}}^{2}}_{\text{x}}\times {{\text{h}}^{2}}_{\text{Y}}\right)\times \text{r}{\text{g}}_{\text{XY}}\times \sigma {\text{p}}_{\text{Y}}$$

where CR_(Y.X) _is the expected correlated response of trait Y when selection in on X; CR_(Y.Y) _is the expected direct response of the selection on Y; i_X _and i_Y _are the intensity of selection on X and Y, respectively; h²_X _and h²_Y _are the heritability estimates for X and Y, respectively; r_gXY _is the genetic correlation between X and Y; and σp_Y _is the standard deviation of Y phenotype. A similar value of 1 was set for i_X _and i_Y_. Since σp_Y _is constant between equations [5] and [6], there were dropped from calculations. Expected responses to selection were thus expressed in units of phenotypic standard deviation.

## Results

### Between line differences

Descriptive statistics for excretion traits are reported in Table 1 for both lines. A line effect was highly significant for all traits. The coefficient of the digestive utilization of dry matter (CDUDM) was 28.2% higher in D+ than in D- birds.

**Table 1:** Basic statistics (LS Means ± Standard Error) for all traits analysed (N ranging from 481 to 602 according to the trait).

Traits	D+	D-	D+/D- ratio (%)	Significance of line effect
BW23	490 ± 3.62	428 ± 3.62	14.5	< 0.001
WG	166 ± 1.69	146 ± 1.69	13.7	< 0.001
FI	285 ± 3.13	363 ± 3.18	-21.5	< 0.001
FCR	1.72 ± 0.03	2.72 ± 0.03	-36.8	< 0.001
CDUDM	75.4 ± 0.56	58.8 ± 0.56	28.2	< 0.001
FEW	245 ± 11.7	417 ± 11.8	-41.3	< 0.001
DEW	70.3 ± 3.27	153 ± 3.29	-54.1	< 0.001
FEW/BW	0.53 ± 0.04	1.04 ± 0.04	-49.0	< 0.001
DEW/BW	0.13 ± 0.01	0.33 ± 0.01	-60.6	< 0.001
FEW/FI	0.85 ± 0.03	1.16 ± 0.03	-26.7	< 0.001
WE	71.0 ± 0.53	65.8 ± 0.53	7.90	< 0.001
NE/NI	0.41 ± 0.01	0.63 ± 0.01	-34.9	< 0.001
PE/PI	0.47 ± 0.01	0.58 ± 0.01	-19.0	< 0.001
NE/PE	3.46 ± 0.04	4.34 ± 0.04	-20.3	< 0.001

^1 ^BW23, body weight at 23 d of age (g); WG, body weight gain between 17 and 23 d of age (g); FI, feed intake between 17 and 23 d of age (g); FCR, feed conversion ratio between 17 and 23 d (g.g^-1^); CDUDM, coefficient of digestive use of dry matter (g); FEW, fresh excreta weight (g); DEW, dry excreta weight (g); FEW/BW and DEW/BW, fresh and dry excreta weight relative to body weight at 23 d; FEW/FI, fresh excreta weight relative to feed intake(g.g^-1^); WE, water content of excreta (%); NE/PE, ratio of nitrogen to phosphorus excretion (g.g^-1^); NE/NI, PE/PI, nitrogen and phosphorus excreted relative to nitrogen and phosphorus intake (g.g^-1^)

Whatever the trait, the D+ birds excreted significantly less than the D- birds. In terms of quantity, FEW and DEW were 70.2% and 118.3% higher in D- birds, respectively. The D+ birds also excreted 35.1% less water than D- birds. This difference partly reflected a difference in feed consumption, which was 27.2% higher in D- birds between 17 and 23d. However, even when correcting for this difference in feed consumption, FEW, DEW, and the gross quantity of water were still 36.8, 67.9, and 25.1% higher in D- birds. Furthermore, the D+ birds excreted 49.0 and 60.6% less fresh and dry excreta than D- birds for the same BW at 23 d of age. In terms of the composition of excreta, the relative nitrogen and phosphorus excretion levels were 34.9 and 19.0% lower for D+ than for D- birds, respectively. As the difference between lines was greater for nitrogen than for phosphorus, the nitrogen to phosphorus ratio in excreta was 20.3% higher for D+ than for D- birds. .

### Heritability estimates for excretion traits

Heritability estimates of BW23, WG, AMEn, FCR, RFI, FI and the gastro-intestinal tract can be found in de Verdal et al. [15]. The heritability estimates of excretion traits are shown in Table 2. Heritability was low for WE and FEW/BW (0.10 and 0.09, respectively). For other excretion traits, estimates were moderate (0.18 to 0.22 for DEW/BW, FEW/FI, NE/PE, and PE/PI). The highest estimates were found for CDUDM and NE/NI (0.29 and 0.30, respectively). CDUDM was also found to be affected by a significant maternal permanent environment effect (0.08 ± 0.01).

**Table 2:** Estimated heritability (± standard errors, on diagonal) and genetic correlations (± standard errors, above diagonal) for excretion traits.

	CDUDM^1^	FEW/BW	DEW/BW	FEW/FI	WE	NE/NI	PE/PI	NE/PE
CDUDM	0.30 ± 0.02	-0.94 ± ne	-0.93 ± ne	-0.64 ± ne2	0.39 ± ne	-1.00 ± ne	-0.68 ± 0.07	-0.87 ± ne
FEW/BW		0.09 ± 0.01	1.00 ± 0.01	0.76 ± 0.07	-0.22 ± 0.25	0.82 ± ne	0.43 ± 0.18	0.76 ± 0.23
DEW/BW			0.20 ± 0.03	0.51 ± 0.09	-0.37 ± 0.15	0.76 ± ne	0.36 ± 0.08	0.67 ± 0.13
FEW/FI				0.17 ± 0.04	0.54 ± ne	0.67 ± ne	0.87 ± 0.08	-0.05 ± 0.17
WE					0.13 ± 0.05	-0.34 ± ne	0.33 ± 0.17	-0.75 ± 0.12
NE/NI						0.29 ± 0.02	0.74 ± 0.06	0.58 ± ne
PE/PI							0.22 ± 0.04	-0.11 ± ne
NE/PE								0.18 ± 0.04

^1 ^CDUDM, coefficient of digestive use of dry matter; FEW/BW and DEW/BW, fresh and dry excreta weight relative to body weight at 23 d; FEW/FI, fresh excreta weight relative to feed intake, WE, water content of excreta, NE/PE, ratio of nitrogen to phosphorus excretion; NE/NI, PE/PI, nitrogen and phosphorus excreted relative to nitrogen and phosphorus intake

^2 ^ne: not estimated

### Genetic correlations between excretion traits

The genetic correlations between the various excretion traits are shown in Table 2. As several traits presented very strong genetic correlations, convergence was more difficult to establish in some analyses, meaning that it was impossible to estimate standard errors of genetic correlations. As expected, CDUDM was highly negatively correlated with all excretion traits, with correlations ranging between -0.64 and -1.00, however the -1.00 correlation between CDUDM and NE/NI was probably an overestimation due to the presence of CDUDM and AMEn in the same analysis. Consistent with this, fresh excreta weight relative to feed intake was highly positively correlated with WE, NE/NI, and PE/PI (between 0.54 and 0.87) but only the latter was significantly different from 0 as standard errors could not be estimated for the first 2 values. The only difference between relative fresh excreta weight and CDUDM was that the former was not genetically correlated with NE/PE (r_g _= -0.05) whereas the latter was very highly correlated with this trait (r_g _= -0.87). The genetic correlation between FEW/BW or DEW/BW and FEW/FI, NE/NI, PE/PI and NE/PE were high (ranging from 0.36 to 0.82). Excretion of nitrogen and phosphorus was highly correlated (r_g _= 0.74). Finally, it should be noted that the balance between N and P in excreta was mainly correlated with N excretion (r_g _= 0.58), but very poorly with P excretion (r_g _= -0.11) but standard errors of the parameters could not be estimated.

### Genetic correlations between excretion and performance traits

The genetic correlations between excretion traits and performance traits are shown in Table 3. AMEn and FCR showed similar correlations with excretion traits, but logically with opposite signs. Except for WE, the correlations with all excretion traits were very high, absolute values of genetic correlations ranging between 0.64 and 0.99. Once again, correlation above 0.97 were obtained in analyses including simultaneously AMEn and CDUDM, very strongly correlated, and/or FEW/BW which has low heritability. In contrast, BW23 and RFI were more moderately correlated with FEW/FI and NE/PE and RFI was correlated with NE/NI but both BW23 and RFI were not correlated with PE/PI. Finally, RFI was highly correlated with FEW/BW and DEW/BW, and BW23 highly correlated with WE. The genetic correlations between FI and the excretion traits were of opposite sign but lower than those between AMEn and the excretion traits.

**Table 3:** Genetic correlations (± standard errors) between excretion traits and performance

Trait^1^	CDUDM	FEW/BW	DEW/BW	FEW/FI	WE	NE/NI	PE/PI	NE/PE
BW23	0.16 ± 0.06	-0.16 ± 0.21	-0.20 ± 0.15	0.44 ± 0.16	0.86 ± 0.10	-0.19 ± 0.12	0.03 ± 0.19	-0.52 ± 0.18
WG	0.21 ± ne	-0.28 ± ne	-0.29 ± ne	0.02 ± ne	0.24 ± 0.22	-0.37 ± ne	-0.42 ± 0.15	-0.16 ± ne
FI	-0.75 ± ne	0.58 ± ne	0.73 ± ne	0.41 ± ne	-0.17 ± 0.18	0.45 ± ne	0.20 ± 0.14	0.34 ± 0.16
FCR	-0.98 ± 0.01	0.99 ± 0.03	0.92 ± 0.05	0.76 ± ne^2^	-0.27 ± ne	0.95 ± ne	0.66 ± 0.11	0.88 ± ne
AMEn	0.99 ± 0.00	-0.97 ± 0.04	-0.92 ± 0.03	-0.66 ± 0.07	0.46 ± 0.15	-0.99 ± 0.01	-0.64 ± 0.07	-0.84 ± 0.08
RFI	-0.64 ± ne	0.91 ± ne	0.88 ± ne	0.38 ± 0.15	-0.15 ± ne	0.37 ± 0.09	0.08 ± 0.14	0.30 ± ne

^1 ^BW23, body weight at 23 d; WG, weight gain between 17 and 23 d; FI, feed intake between 17 and 23 d; FCR; feed conversion ratio; AMEn, apparent metabolisable energy corrected for zero nitrogen balance; RFI, residual feed intake; CDUDM, coefficient of digestive use of dry matter; FEW/BW and DEW/BW, fresh and dry excreta weight relative to body weight at 23 d; FEW/FI, fresh excreta weight relative to feed intake; WE, water content of excreta; NE/NI, PE/PI, ratio of nitrogen and phosphorus excretion to intake; NE/PE, ratio of nitrogen to phosphorus excretion.

^2 ^ne: not estimated

The expected response on excretion traits to direct selection or to indirect selection of AMEn, CDUDM, FCR and RFI are shown in Table 4. The values were calculated with the equations [5] and [6], supposing that selection intensity was 1 for all traits. Direct selection on excretion traits showed lower or rather similar expected responses than indirect selection on AMEn or CDUDM. Moreover, the expected responses of a selection on feed efficiency were similar or lower than direct, AMEn or CDUDM selection, except for the indirect expected response of a RFI selection for DEW/BW and FEW/BW.

**Table 4:** Expected responses to direct selection on excretion traits or on indirect selection on digestibility (AMEn and CDUDM) and on feed efficiency (FCR and RFI), supposing that selection intensity was 1 for all traits.

		Selection on			
		
Response^1^	Direct	AMEn	CDUDM	FCR	RFI
CDU-DM	0.300	0.297	0.300	-0.246	-0.238
FEW/BW	0.090	-0.159	-0.154	0.136	0.185
DEW/BW	0.200	-0.225	-0.228	0.189	0.267
FEW/FI	0.170	-0.149	-0.145	0.144	0.106
WE	0.130	0.091	0.077	-0.045	-0.037
NE/NI	0.290	-0.292	-0.295	0.234	0.135
PE/PI	0.220	-0.164	-0.175	0.142	0.025
NE/PE	0.180	-0.195	-0.202	0.171	0.086

Responses are expressed in phenotypic standard deviations of the trait.

^1^AMEn, apparent metabolisable energy corrected for zero nitrogen balance; CDUDM, coefficient of digestive use of dry matter; FCR, feed conversion ratio; RFI, residual feed intake; FEW/BW, DEW/BW, fresh and dry excreta weight relative to body weight at 23 d; FEW/FI, fresh excreta weight relative to feed intake; WE, water content of excreta; NE/NI, PE/PI, ratio of nitrogen and phosphorus excretion to intake; NE/PE, ratio of nitrogen to phosphorus excretion

### Genetic correlation between excretion traits and GIT morphology

The genetic correlations between the excretion traits and GIT characteristics are shown in Table 5. All the GIT organs were correlated with excretion traits. CDUDM was positively correlated with relative proventriculus and gizzard weights (0.63 and 0.43, respectively), and negatively correlated with the relative weight and the density of the intestinal segments (correlations ranging from -0.35 to -0.75) but not with their relative length. It is however to note that standard errors were not available for genetic correlations between CDUDM and PRW, JW, and ID (0.63, -0.66 and -0.52, respectively). In contrast, the fresh excreta weight relative to feed intake was positively correlated only with the relative weight of the ileum (r_g _= 0.47) and with the density of the 3 intestinal segments (from 0.42 to 0.72). An increased water excretion rate was genetically linked to a shorter and denser intestine (correlations ranging from 0.45 to 0.90 in absolute values), and with a lighter proventriculus (r_g _= -0.48).

**Table 5:** Genetic correlations (± standard errors) between excretion traits and gastro-intestinal tract morphology

Trait^1^	CDUDM	FEW/BW	DEW/BW	FEW/FI	WE	NE/NI	PE/PI	NE/PE
CW	0.11 ± 0.10	0.43 ± 0.17	0.17 ± 0.15	0.30 ± 0.19	0.26 ± ne	-0.11 ± 0.13	0.02 ± 0.09	-0.13 ± ne
PRW	0.63 ± ne	-0.44 ± 0.32	0.02 ± 0.22	0.04 ± 0.18	-0.48 ± 0.18	-0.12 ± 0.19	0.36 ± 0.18	-0.48 ± 0.18
GZW	0.43 ± 0.15	0.01 ± 0.20	-0.19 ± 0.15	-0.14 ± ne	0.13 ± 0.21	-0.20 ± 0.14	0.07 ± 0.18	-0.23 ± 0.20
DW	-0.37 ± 0.08	0.44 ± 0.17	0.32 ± 0.10	-0.02 ± 0.15	-0.48 ± ne	0.42 ± 0.10	0.20 ± 0.15	0.05 ± 0.14
JW	-0.66 ± ne	0.65 ± 0.10	0.50 ± 0.11	0.17 ± ne	-0.38 ± 0.20	0.72 ± 0.08	0.63 ± 0.12	0.28 ± 0.14
IW	-0.75 ± 0.07	0.73 ± 0.10	0.44 ± ne	0.47 ± 0.15	0.09 ± 0.16	0.70 ± 0.06	0.76 ± 0.11	0.20 ± 0.14
DL	-0.03 ± ne	0.02 ± ne	0.17 ± ne	-0.35 ± 0.18	-0.87 ± 0.07	0.19 ± ne	-0.09 ± ne	0.54 ± ne
JL	-0.06 ± ne	-0.19 ± 0.19	0.09 ± 0.12	-0.25 ± 0.19	-0.90 ± 0.09	0.25 ± ne	0.17 ± 0.17	0.37 ± 0.18
IL	-0.01 ± ne	-0.20 ± ne	0.06 ± 0.13	-0.31 ± 0.19	-0.77 ± 0.21	0.08 ± ne	0.07 ± ne	0.23 ± ne
DD	-0.35 ± 0.08	0.43 ± 0.16	0.10 ± 0.12	0.48 ± 0.15	0.54 ± 0.22	0.28 ± 0.10	0.33 ± 0.15	-0.10 ± 0.16
JD	-0.41 ± 0.08	0.59 ± 0.15	0.25 ± 0.12	0.42 ± 0.14	0.45 ± 0.20	0.32 ± ne	0.39 ± 0.14	-0.14 ± 0.12
ID	-0.52 ± ne	0.68 ± ne	0.27 ± 0.13	0.72 ± 0.13	0.55 ± ne	0.50 ± 0.10	0.57 ± 0.13	-0.14 ± 0.14

^1^CW, PRW, GZW, LW, DW, JW, IW, relative weights of crop, proventriculus, gizzard, liver, duodenum, jejunum, and ileum; DL, JL, IL, relative lengths of duodenum, jejunum, and ileum; DD, JD, ID, density of duodenum, jejunum, and ileum; CDUDM, coefficient of digestive use of dry matter; FEW/BW and DEW/BW, fresh and dry excreta weight relative to body weight at 23 d; FEW/FI, fresh excreta weight relative to feed intake; WE, water content of excreta; NE/NI, PE/PI, ratio of nitrogen and phosphorus excretion to intake; NE/PE, ratio of nitrogen to phosphorus excretion.

^2 ^ne: not estimated

A high positive genetic correlation was observed between phosphorus and nitrogen excretion and relative weights of jejunum and ileum (r_g _between 0.63 and 0.76), but only moderate correlations with densities (r_g _= 0.39 on average). In the same way, FEW/BW and DEW/BW were positively correlated with intestine relative weight and density, the only non significant correlation being found between DEW/BW and DD. Phosphorus excretion was also moderately correlated with PRW, and nitrogen excretion with duodenum weight relative to BW23. The similarity of genetic correlations of anatomic traits with NE/NI and PE/PI ratios mean that the NE/PE ratio was weakly or moderately correlated with anatomic characteristics. Indeed, the NE/PE ratio was only moderately correlated with proventriculus relative weight and with jejunum relative weight and length.

## Discussion

### Heritability estimates of excretion traits

Genetic parameters of digestibility, feed efficiency and anatomy of the digestive tract have been discussed previously by de Verdal et al. [15] on the same data set and are not detailed further here. However, it should be noted that the D+ birds had 33.5% higher AMEn, 14.5% higher BW23 and 36.8% lower FCR than D- birds. Furthermore, AMEn and FCR heritabilities were estimated at 0.30 and 0.21, respectively [15].

While chicken manure can be used as fertiliser, at high levels it is considered a pollutant, increasing water eutrophication, excessive algae development and ammonia volatilisation in the air. Thus, in view of the problems related to the management and the environmental impact of chicken manure, the selection of birds producing reduced quantities of excreta is important.

To our knowledge, the present study is the first to present estimated genetic parameters of broiler excretion traits and their correlations with performance characteristics and GIT morphology. However, probably due to the low number of birds used, the standard errors were sometimes relatively high, and consequently, some results should be taken with caution. In some other cases, several parameters in the same analysis were close to the limit of parameter space (genetic correlation close to unity and h² of some traits close to 0), which makes convergence more difficult. It was for example the case when FEW/BW with low h² was included simultaneously with DEW/BW, with which it was very highly correlated. Similarly, even if FCR and AMEn had already been shown to be strongly correlated (-0.70 [10]), the genetic correlation of 0.98 between CDUDM and FCR was probably overestimated due to the presence of 3 highly correlated traits in the analysis.

Excretion traits were moderately heritable, showing that it should be possible to include such traits in poultry selection. The estimated heritability of PE was much higher than that reported by Zhang et al. [23] and Ankra-Badu et al. [24] who reported a value of 0.09 for phytic phosphorus bioavailability (PBA). However, even with this rather low heritability, Zhang et al. [8] obtained a divergence of 9.7% on P bioavailability after 3 generations. We can hypothesize that this wide difference between different studies is related to the diet used, as Zhang et al. [23] and Ankra-Badu et al. [24] used a corn-based diet that is easy to digest, while the wheat diet used in the present study made it easier to distinguish between animals with poor or high capacity of retention. Furthermore, these experiments differed from ours by genetic lines used, which showed a much slower growth than ours. Mignon-Grasteau et al. [25] showed that heritability estimates of metabolisable energy and coefficients of digestive use of proteins and lipids were much higher when animals were fed with poor wheat than with corn.

### Phenotype differences between D+ and D- lines and genetic correlations for excretion traits

In the present study, we found that D+ birds had a 28.2% greater CDUDM than D- birds, showing that digestive utilization was improved in D+ compared to D- birds. This could be explained by the genetic correlations between CDUDM and GIT morphology. Indeed, it seems that selection on high CDUDM would increase the relative weight of the upper part of the GIT (proventriculus and gizzard) and conversely decrease the relative weight and the density of the small intestine, consistent with previous results [12]. A larger gizzard and proventriculus would lead to greater nutrient accessibility in the small intestine and thus to better digestive efficiency. At the intestinal level, the genetic correlations were higher between CDUDM and the relative weights of the jejunum and the ileum than between the CDUDM and the relative weight of the duodenum, which could potentially be explained by the fact that absorption processes mainly take place in the jejunum and ileum [26].

This higher digestive utilisation in D+ birds leads to a 41.3 and 54.1% reduction in FEW and DEW, respectively, compared to D-. These differences are also present at later ages. Furthermore, the commercial line used at the beginning of the selection experiment excreted 31.5% more DEW/FI between 21 and 53 d of age than D+ birds [11]. Selection for a better AMEn thus led to a reduced environmental impact of chicken production. Although WE was greater in D+ birds, the total quantity of water excreted and the FEW/FI ratio were 51.2% and 26.7% lower than in D- birds. As Williams et al. [27] explained that water consumption closely follows food consumption, and since FI was 27.4% higher in D- than in D+ birds, it could be hypothesized that D- birds consume almost 30% more water than D+ birds. This is important since this can have consequences in terms of health and welfare. An increase in the quantity of water excreted would lead to a more humid litter and consequently to an increase in the incidence of associated poultry diseases, such as breast blisters, skin burns, scabby areas, bruising, rejection or downgrades [28]. Moreover, the litter moisture content is known to have a high impact on the ammonia losses by volatilization, which may cause respiratory disorders in birds and farmers and increase the imbalance between N and P in manure [29].

However, most of the studies related to environmental problems due to the spreading of manure focus on N and P content [30] and their deleterious environmental impact. The capacity of D+ birds to retain N and P was 34.9 and 19.0% higher, respectively, compared to the D-, as shown by NE/NI and PE/PI ratios. Thus, for each 100 g of BW, D+ and D- excreted 0.73 and 1.65 g of N and 0.17 and 0.32 g of P, respectively. It has already been shown that the lower NE/NI ratio in D+ can be linked to the 8.7 to 13.1% better ability of these birds to utilise proteins [10,13,25]. Moreover, the NE/NI ratio was more highly correlated genetically with the lower rather than the upper part of the GIT. This suggests a major contribution of the lower part of the intestine compared to the upper part in N utilization. Péron et al. [31] showed that the pancreas was heavier in D- than in D+ birds, and found negative phenotype correlations between pancreas weight in relation to BW and AMEn and lipid, protein and starch digestibility. These authors explained that the enlargement of the pancreas could be an adaptation to decreased digestion in D- birds.

Furthermore, NE/NI and PE/PI ratios were more genetically correlated with jejunum and ileum relative weights and densities than with those of the duodenum. This illustrates the major contribution of the lower part of the intestine in N and P absorption [32,33]. Nevertheless, in contrast to the NE/NI ratio, the PE/PI ratio was positively genetically correlated with PRW, indicating a major contribution of this segment to P availability. These results are probably related to the morphological and functional differences in the upper GIT characterizing both lines [12,34]. Indeed, the greater development of the upper part of the GIT in D+ birds may underlie an increase in the synthesis of hydrochloric acid. Moreover, the mean retention time in the upper part of the GIT is greater in D+ than in D- birds [35]. All of these phenomena could lead to a lower pH of digesta that promote solubility of mineral phosphates [36] in D+ birds and the capacity of residual endogenous phytase of the feed [37]. They can also favour the hydrolysis of phytic P by endogenous bacteria [38].

These high levels of differences in N and P excretion between D+ and D- birds could explain why the ratio of the NE to PE was 25.4% higher in D- than in D+ birds. French and European regulations limit the amounts of N and phosphates (P_2_O_5_) that can be spread on fields to 170 kg. ha^-1 ^and 100 kg.ha^-1^, respectively, the ideal ratio of N to P_2_O_5 _on spread manure should thus be 1.7 [2]. Considering the litter and the water part of the manure being spread, the ratio of N to P_2_O_5 _that would be found in the manure would be 1.95 and 2.33 for the D+ and the D- birds, respectively. However, since 50% of the N excreted by chickens is lost between excretion and spreading [39], these ratios would become 0.976 for the D+ and 1.166 for the D- birds for manure ready to be spread on fields, implying that the manure of both lines is too rich in P_2_O_5 _compared to N. This suggests first that N losses should be limited to increase the N/P_2_O_5 _ratio in manure and secondly that this limitation should take into account the genotype of birds. Indeed, N losses in manure should be limited to 15% in D+ birds and 37% in D-birds, whereas the usual value is closer to 50%.

A second way to improve manure quality would be to combine genetic and nutritional approaches, i.e. by reducing the P rate in the diet and adjusting the phytase quantity added to the diet to each genotype in order to reduce P excretion [40].

### Direct selection on excretion traits *vs *indirect selection on efficiency

It is often assumed that excretion can be reduced by selection on feed efficiency. By providing a full set of genetic parameters of excretion traits and efficiency, our study allows comparison of the expected responses to direct selection on excretion traits and digestibility and to indirect selection on feed efficiency. Using equations [5] and [6], for most excretion traits (CDUDM, WE, NE/NI, PE/PI and NE/PE) the expected responses to selection on FCR would be reduced by 12.3 to 50.5% as compared to selection on AMEn or CDUDM, and selection on RFI would lead to expected responses reduced by 19.9 to 85.7% compared to AMEn or CDUDM selection. While the indirect expected response was higher for selection on AMEn or CDUDM than on FCR for DEW/BW and FEW/BW, a selection on RFI would be 16.4 to 20.1% more efficient than on AMEn or CDUDM. At the opposite, for the FEW/FI ratio, the expected responses were similar for selection on AMEn, CDUDM or FCR, but selection on RFI would be 26.4 to 28.9% less efficient.

It therefore appears that, in order to reduce environmental pollution, selecting chickens on AMEn or CDUDM would be more effective than selection on feed efficiency, all the more true that actual methods (as NIRS) allow measuring these traits at a very moderate cost.

Besides AMEn or CDUDM selection, direct selection on excretion traits could be considered. Using equation [5], it appears that for FEW/FI, DEW/FI, NE/PE and NE/NI ratios, indirect selection on AMEn or CDUDM would be more effective than direct selection, with improvements ranging from 1 to 78%. For the other excretion traits, indirect selection could be almost as effective as direct selection. Indeed, the responses of the FEW/FI ratio to indirect selection on AMEn or CDUDM were 88.2% of those of direct selection. Similarly, using AMEn or CDUDM as indirect selection criterion of WE would also be very effective (ranging from 62 to 69% of the direct response). Moreover, the PE/PI ratio would be considerably modified by selection on AMEn or CDUDM, with indirect responses ranging between 73 and 82% of the direct response. Consequently, introducing AMEn or CDUDM in selection schemes could be a good way to reduce excretion and hence the environmental impact of chicken production. Finally, if evolution of genetic values in D+ and D- are symmetric, it is not the case for phenotypic values, which is commonly observed in divergent selection experiments. To draw a definitive conclusion on practical interest of such a selection, it would be necessary to compare to a control line (CL) such as the line used at the beginning of selection experiment. First elements brought by such a comparison indicated that DEW/FI was 31.5% lower in D+ than in CL birds [11], between 21 and 53 d (age at which birds reached commercial market weight).

## Conclusion

Our genetic results indicate that limiting the environmental impact of chicken production by selection could be achieved by selecting on AMEn as well as on the CDUDM. According to the estimated genetic correlations, a decrease in chicken excretion is associated with an increase in proventriculus and gizzard relative weights, which would be likely to improve nutrient accessibility in the small intestine and thus the digestibility. Because of the increased competition between humans and animals for access to food (mainly cereals) and the use of non-renewable materials (such as inorganic P) in animal nutrition, the adaptation of birds to alternative diets of lower nutritional quality will become an important issue. This study highlights that there is wide genetic variability, and this may be used to improve feed digestibility and thus limit the excretion responsible for environmental pollution. Finally, even if classical selection criteria as FCR would reduce environmental impact of poultry production, greater responses could be expected from selection on digestive efficiency.

## Competing interests

The authors declare that they have no competing interests

## Authors' contributions

HDV, AN, ELB and SMG contributed to the experimental design, data analysis, interpretation of data and manuscript preparation. HC contributed to the data analysis. DB contributed to the use of NIRS in digestibility measurements. NM and SU assisted in the acquisition of data. All authors read and approved the final manuscript.
